# A comparison of the Indian diet with the EAT-Lancet reference diet

**DOI:** 10.1186/s12889-020-08951-8

**Published:** 2020-05-29

**Authors:** Manika Sharma, Avinash Kishore, Devesh Roy, Kuhu Joshi

**Affiliations:** International Food Policy Research Institute, NASC Complex, CG Block, Dev Prakash Shastri Marg, Pusa, New Delhi, 110012 India

**Keywords:** Diet, Protein, Processed food, India, NSS, Calories, Consumption, EAT-Lancet, Reference diet, Food system

## Abstract

**Background:**

The 2019 EAT-Lancet Commission report recommends healthy diets that can feed 10 billion people by 2050 from environmentally sustainable food systems. This study compares food consumption patterns in India, from different income groups, regions and sectors (rural/urban), with the EAT-Lancet reference diet and highlights the deviations.

**Methods:**

The analysis was done using data from the Consumption Expenditure Survey (CES) of a nationally representative sample of 0.102 million households from 7469 villages and 5268 urban blocks of India conducted by the National Sample Survey Organization (NSSO) in 2011–12. This is the most recent nationally representative data on household consumption in India. Calorie consumption (kcal/capita/day) of each food group was calculated using the quantity of consumption from the data and nutritional values of food items provided by NSSO. Diets for rural and urban, poor and rich households across different regions were compared with EAT-Lancet reference diet.

**Results:**

The average daily calorie consumption in India is below the recommended 2503 kcal/capita/day across all groups compared, except for the richest 5% of the population. Calorie share of whole grains is significantly higher than the EAT-Lancet recommendations while those of fruits, vegetables, legumes, meat, fish and eggs are significantly lower. The share of calories from protein sources is only 6–8% in India compared to 29% in the reference diet. The imbalance is highest for the households in the lowest decile of consumption expenditure, but even the richest households in India do not consume adequate amounts of fruits, vegetables and non-cereal proteins in their diets. An average Indian household consumes more calories from processed foods than fruits.

**Conclusions:**

Indian diets, across states and income groups, are unhealthy. Indians also consume excess amounts of cereals and not enough proteins, fruits, and vegetables. Importantly, unlike many countries, excess consumption of animal protein is not a problem in India. Indian policymakers need to accelerate food-system-wide efforts to make healthier and sustainable diets more affordable, accessible and acceptable.

## Background

A healthy diet is key for optimal nutrition and health outcomes through all stages of the lifecycle. Unhealthy diets are linked to all forms of malnutrition and various diseases [1]. World Health Organization (WHO) recognizes unhealthy diets along with inadequate physical activity as one of the risk factors for non-communicable diseases. High fat intake, low fruit and vegetable intake, overweight and obesity, physical inactivity, raised blood glucose, raised blood pressure, raised total cholesterol, high salt/sodium intake are amongst the exposures that lead to non-communicable diseases (NCDs) [2]. The prevalence of obesity and NCDs has been rising across the world [3–5] even as undernutrition and communicable disease burden remains high.

An analysis of India’s disease burden from 1990 to 2016 showed that heart diseases cause the most deaths in India while dietary iron deficiency is the biggest contributor to disability [6]. High prevalence of both anaemia and heart diseases shows the rising problem of the double burden of undernutrition and overnutrition in India. Unhealthy diets are a major contributor to this syndemic.

The global food system is unhealthy not only for humans, but also for the environment. On one hand, our existing diets contribute to multiple forms of malnutrition and the rising incidence of NCDs. On the other hand, food production has a large deleterious impact on multiple environmental variables like freshwater availability, soil quality, forest cover, biodiversity, coastal eutrophication, and climate change. Food, therefore, is as much an environmental issue as it is a health issue. This is the point of departure for the EAT-Lancet Commission. It sets two hard boundaries: first, the quantity and quality of foods and second, the environmental limits or the planetary boundaries. The Lancet Commission report (2019) sets out to answer the following question: What could we eat that would feed 10 billion people in 2050 a healthy diet within the environmental limits.

On the environment front, the commission has set scientific targets for the earth system processes - climate change, nitrogen and phosphorus cycling, freshwater use, biodiversity loss, and land-system change to lay the parameters necessary for sustainable food production [7].

On the consumption side, the report lays out a reference diet for individuals aged 2 years and above with reference range for food groups allowing for flexibility in its application while taking nutritional adequacy into account. It consists largely of plant-based foods: vegetables, fruits, whole grains, legumes, nuts, and unsaturated oils. It includes moderate amounts of seafood and poultry and no or small quantities of red meat, processed meat, added sugar, refined grains and starchy vegetables [7]. The EAT-Lancet Commission report acknowledges the challenges in defining a global reference diet owing to differences in body size, physical activity, disease status and needs of vulnerable populations like pregnant women and young children [7].

The reference diet as proposed is not aimed at providing national targets and allows for flexibility in its application. It does outline, in principle, the healthy diet with its relative food group composition. A big difference between the EAT-Lancet reference diet and the existing recommended dietary allowances (RDAs), like the one by the Indian Council of Medical Research (ICMR), is that the former also takes the environmental footprints of different foods into account while the latter focuses only on the human nutritional requirements. Accounting for environmental footprints makes EAT-Lancet recommend a more vegetarian diet than a typical RDA.

This paper does not discuss the environmental aspect of the reference diet and focusses exclusively on the divergence of the current Indian diets from the composition of the proposed reference diet by EAT-Lancet. From a public health perspective, this can clarify the policy and programmatic changes that might be needed in India for transformation to a healthier diet for better nutrition, health, and environmental outcomes.

## Methods

The EAT-Lancet reference diet is made up of 8 food groups - whole grains, tubers and starchy vegetables, fruits, other vegetables, dairy foods, protein sources, added fats, and added sugars. Caloric intake (kcal/day) limits for each food group are given and add up to a 2500 kcal daily diet [7]. We compare the proportional calorie (daily per capita) shares of the food groups in the reference diet with similar food groups in Indian Diets.

We use data from the most recent round of the household Consumption Expenditure Survey (CES) conducted by the National Sample Survey Organization (NSSO) of India in 2011–12. NSS-CES has been conducted every 5 years from 1972 to 73 onwards. Each quinquennial round covers a nationally representative sample of more than 100 thousand households from both rural and urban areas of all states and union territories of India. The NSS-CES sample is also representative at the state level.^1^

Data is collected from a random sample of households across India. Floating populations, foreign nationals and their domestic servants, soldiers in barracks, and kids in orphanages are excluded from the survey. This sample covers all kinds of households. A random 25% subsample of the total sampled households from each district of India are surveyed in all 4 quarters of the year to account for any seasonal variations in consumption patterns and obtain a more representative data of the usual intakes of households.

It is the primary source of public data on various indicators of the level of living of different segments of the population at national and state levels in India. This data has been extensively used for planning and policy formulation by government organizations, academicians, researchers, and scholars.

NSS-CES collects both 30-day and 7-day recall data on the quantity of consumption and the market value of 147 different food items by each household in the sample [8]. Detailed list of items in the expenditure survey helps get more accurate data. 30-day recall data is collected for cereals, legumes, milk & milk products, sugar, and salt while 7-day recall for edible oil, egg, fish, meat, vegetables, fruits, spices, beverages, and processed foods. The survey also records the value of purchased snacks and the number of meals eaten out of the home by household members.

We aggregated each of the 147 food items into 10 food groups. Eight food groups like the EAT-Lancet reference diet and two additional food groups: processed foods and spices. We included these two additional food groups because the consumption of processed foods is rising rapidly, and spices are an essential part of the Indian diet.

For Indian diets, the food group ‘whole grains’ comprises of cereals like rice, wheat, wheat flour and other cereals like jowar (sorghum), bajra (pearl millet), maize, barley, ragi (finger millets), other millets and other cereals. ‘Vegetables’ include all vegetables except for potatoes. ‘Fruits’ include all fresh fruits and dried fruits like dates, raisins, and other dry fruits. All beans, pulses, and groundnuts have been classified into ‘Legumes’. Food group ‘Tree nuts’ includes coconut, coconut-green, coconut-*copra*, walnuts, cashew nuts, and other nuts.

The food group ‘Added Fats’ in the EAT-Lancet reference diet includes palm oil, unsaturated oils, dairy fats and lard [7]. NSS-CES uses a different classification for edible oils and fats. For comparison with the reference diet, NSS-CES food items *vanaspati* (partially hydrogenated vegetable oil), refined and edible oil have been aggregated as ‘palm oil’ due to their high content of palm and palm olein oil [9] and mustard, groundnut and coconut oil as ‘unsaturated oil’ due to higher content of unsaturated fatty acids. It should be noted that many cooking oils used in India are a mixture of saturated and unsaturated oils which presents a limitation in this type of analysis [9].

The EAT-Lancet commission recommends “low” amounts of processed foods like refined grains, added sugar and highly processed foods. It has not been included as a part of the 2500 kcal reference diet [7]. It is, however, taken as a separate food group for Indian diets in this study as it forms a significant and growing portion of the total caloric intake by Indian households. It consists of processed cereals like *maida,* (refined wheat flour) and semolina along with packaged food like savouries (*namkeens*), chips, chocolates, sugary beverages, and other food consumed outside of the home. Another category of food that is not mentioned in the EAT-Lancet reference diet, but accounts for 1–2% of total calories in Indian diet is ‘spices’. Spices have been mentioned separately for the Indian diets.

The NSS dataset records the quantity of food items consumed by the household. This is given in grams or kilograms for the 30- or 7-day recall period, depending on the food item. We convert household consumption into per capita per day consumption and use sampling weights provided in the NSS dataset to estimate population averages. We then calculate the caloric intake of food items by multiplying per capita per day quantity consumed with the calorie content of each food item. We thus obtain total calorie intake per capita per day (kcal/capita/day) of food items. These are aggregated across the 10 food groups as mentioned above.

Calorie content of food items is taken from the NSS 68th round report based on “Nutritive Values of Indian Foods” by C. Gopalan, B.V. Ramasastry and S. C Balasubramanian, revised and updated by B.S. Narasinga, Y.G. Deosthale and K.C. Pant, 1991 [10].

NSS-CES reports consumption of some food items, like fruits and packaged foods, either in numbers or the money spent on them. This is the reason why we use calorie intake and not quantities consumed to compare the Indian diet with the reference diet. Differences are calculated as the simple difference and the percentage difference between the total calorie intake of the Indian diet and the EAT-Lancet reference diet.

There are large differences in dietary patterns of rural and urban households, rich and poor households, and people living in different parts of India [11]. Therefore, we also compare the EAT-Lancet reference diet with diets across regions, places of residence (urban and rural), and expenditure classes.

## Results

The average calorie intake/person/day in both rural (2214 kcal) and urban (2169 kcal) India is less than the reference diet (Table 1). In both rural and urban areas, people in rich households (top deciles of monthly per capita consumption expenditure (MPCE)) consume more than 3000 kcal/day i.e. 20% more than the reference diet. Their calorie intake/person/day is almost twice as high as their poorest counterparts (households in the bottom MPCE deciles) who consume only 1645 kcals/person/day (Table 1).

**Table 1:** Daily per capita caloric intake vis-à-vis EAT-Lancet reference diet

	Total Caloric Intake in kcal (95% CI)	% difference
Urban India	2169 (2160, 2179)	−13%
Rural India	2214 (2202, 2225)	−12%
Highest MPCE – Urban	3079 (3010, 3149)	+ 23%
Highest MPCE – Rural	3174 (3024, 3323)	+ 27%
Lowest MPCE – Urban	1643 (1620, 1667)	−34%
Lowest MPCE – Rural	1645 (1616, 1674)	−34%
North India	2259 (2241, 2277)	−10%
South India	2171 (2156, 2186)	−13%
East India	2211 (2194, 2227)	−12%
West India	2124 (2099, 2149)	−15%
North East India	2158 (2119, 2198)	−14%
Central India	2198 (2170, 2226)	−12%

Values in kcal and as % difference with the reference diet (2503 kcal). 95% confidence interval in parenthesis. NSS-CES sampling weights were applied in calculations

North: Delhi, Haryana, Himachal Pradesh, J&K, Punjab, Rajasthan, Uttar Pradesh and Uttarakhand Chandigarh

South: Andhra Pradesh, Kerala, Tamil Nadu, Puducherry, Karnataka and Lakshadweep (Telangana included within data for Andhra Pradesh as NSS 68th round data was collected prior to the separation of the two states)

East: Bihar, Jharkhand, Odisha, West Bengal and A&N Island

West: Goa, Gujarat, Maharashtra, D&N Haveli and Daman&Diu

North East: Arunachal Pradesh, Assam, Manipur, Meghalaya, Mizoram, Nagaland, Sikkim and Tripura

Central: Chhattisgarh and Madhya Pradesh

Why obesity is rising in India despite a lower average total calorie intake, as compared to the EAT-Lancet reference diet or even the ICMR recommendations,^2^ is a question beyond the scope of this paper. However, it may be related to the fact that the reference diet is developed for individuals with moderate-to-high levels of activity, whereas available studies estimate high levels of inactivity amongst Indians. A Lancet study looking at trends of insufficient physical activity between 2001 and 2016 suggested that 34% of Indians are not sufficiently engaged in physical activity [12]. Similarly, a 2014 ICMR-INDIAB study indicates that more than half of all Indians are inactive. Lack of adequate physical activity is found to be more prevalent in urban areas and amongst females [13].

### Calorie shares of different food groups

A healthy diet is diverse and has a balance in calorie shares of different food groups. Tables 2 and 3 show that except for the richest households in urban areas, whole grains (cereals) account for a very high share of total calories consumed by most Indian households. Indians also consume more starchy vegetables, dairy foods, and palm oil as compared to the reference diet. Consumption of processed foods is also high in India, especially among the richest urban households.

**Table 2:** Daily per capita caloric intake comparison by food groups – across sectors and MPCE deciles

	EAT-Lancet	Sectors		Highest MPCE Decile		Lowest MPCE Decile	
		Urban	Rural	Urban	Rural	Urban	Rural
Whole grains^a^	811 (32%)	1029 (1023, 1034) (47%)	1275 (1269, 1282) (58%)	864 (831, 897) (28%)	1347 (1292, 1403) (42%)	1069 (1047, 1091) (65%)	1159 (1132, 1185) (70%)
Potato and Cassava	39 (2%)	51 (51, 52) (2%)	63 (62, 64) (3%)	56 (52, 60) (2%)	58 (53, 62) (2%)	56 (53, 59) (3%)	57 (54, 61) (3%)
Vegetables	78 (3%)	63 (62, 63) (3%)	53 (53, 54) (2%)	93 (89, 97) (3%)	90 (83, 97) (3%)	35 (34, 36) (2%)	30 (29, 31) (2%)
Fruits^b^	126 (5%)	46 (45, 47) (2%)	28 (27, 28) (1%)	122 (116, 129) (4%)	81 (77, 85) (3%)	9 (8, 10) (1%)	5 (4, 6) -
Dairy Foods	153 (6%)	184 (182, 186) (8%)	145 (143, 148) (7%)	362 (347, 378) (12%)	366 (348, 383) (12%)	49 (45, 52) (3%)	27 (24, 30) (2%)
Protein sources	726 (29%)	151 (149, 153) (7%)	125 (124, 127) (6%)	230 (219, 241) (7%)	262 (246, 277) (8%)	76 (74, 78) (5%)	63 (61, 66) (4%)
All Animal source proteins	151 (6%)	33 (33, 34) (2%)	26 (25, 26) (1%)	60 (54, 65) (2%)	66 (60, 72) (2%)	10 (9, 11) (1%)	6 (5, 7) -
Beef and lamb	15 (1%)	5 (5, 5) -	3 (3, 3) -	7 (6, 9) -	11 (8, 14) -	2 (1, 2) -	0 (0, 0) -
Pork	15 (1%)	0 (0, 0) -	0 (0, 0) -	0 (0, 0) -	0 (0, 0) -	0 (−1, 0) -	0 (0, 0) -
Poultry (Chicken & other)	62 (2%)	8 (8, 9) -	6 (6, 6) -	16 (14, 19) (1%)	16 (13, 20) (1%)	1 (1, 2) -	1 (0, 1) -
Eggs	19 (1%)	10 (10, 10) -	6 (6, 6) -	18 (16, 20) (1%)	13 (12, 14) -	3 (2, 4) -	1 (1, 2) -
Fish	40 (2%)	8 (8, 9) -	9 (9, 9) -	16 (14, 18) (1%)	23 (21, 26) (1%)	3 (2, 3) -	2 (2, 3) -
Legumes^c^	426 (17%)	95 (95, 96) (4%)	84 (83, 85) (4%)	122 (117, 127) (4%)	143 (132, 153) (5%)	61 (59, 63) (4%)	55 (53, 58) (4%)
Tree nuts^d^	149 (6%)	21 (21, 22) (1%)	14 (14, 15) (1%)	47 (43, 51) (2%)	52 (48, 56) (2%)	4 (3, 4) -	1 (1, 2) -
Added fats	450 (18%)	271 (269, 274) (12%)	207 (204, 210) (9%)	370 (352, 388) (12%)	375 (316, 434) (12%)	137 (133, 142) (8%)	104 (100, 109) (6%)
Palm Oil^e^	60 (2%)	154 (151, 156) (7%)	94 (91, 97) (4%)	222 (206, 238) (7%)	201 (152, 250) (6%)	54 (49, 60) (3%)	38 (33, 43) (2%)
Unsaturated Fats^f^	354 (14%)	101 (99, 104) (5%)	107 (105, 109) (5%)	104 (94, 115) (3%)	147 (133, 161) (5%)	82 (76, 87) (5%)	65 (61, 70) (4%)
Dairy Fats	0 (0%)	15 (15, 16) (1%)	5 (4, 5) -	42 (39, 46) (1%)	26 (14, 38) (1%)	0 (0, 1) -	0 (−1, 0) -
Lard	36 (1%)	–	–	–	–	–	–
All sweeteners	120 (5%)	114 (113, 115) (5%)	102 (101, 104) (5%)	139 (133, 145) (5%)	202 (178, 227) (6%)	65 (63, 68) (4%)	47 (45, 49) (3%)
Processed Food^g^		263 (256, 270) (12%)	204 (199, 209) (9%)	911 (821, 1001) (30%)	407 (356, 458) (13%)	131 (119, 142) (8%)	136 (123, 149) (8%)
Spices^h^		39 (39, 40) (2%)	35 (34, 35) (2%)	51 (49, 54) (2%)	63 (58, 68) (2%)	21 (21, 22) (1%)	18 (17, 18) (1%)
**Total**	2503	2169	2214	3079	3174	1643	1645

Mean values in kcal reported with 95% confidence intervals in parenthesis. Second row of each cell reports the mean value as a % of daily total calorie consumption. NSS-CES sampling weights were applied in calculations

Lighter font represents subgroups within major food groups

MPCE (Monthly Per Capita Expenditure) signifies classification of population by economic level. Highest and lowest MPCE refers to the richest and poorest 5% of the population respectively

^a^Whole grains consist of cereals like rice, wheat, wheat flour and other cereals like jowar, bajra, maize, barley, millets and ragi

^b^Fruits consists of all fruits and raisins, dates and other dried fruits

^c^Legumes consists of all pulses and groundnuts,

^d^Tree nuts consist of coconut, coconut green and coconut-*copra* (dried), cashew nuts, walnuts and other nuts

^e^Palm oil contains vanaspati, refined and edible oil

^f^Unsaturated oil contains mustard, groundnut and coconut

^g^Processed Food includes rice products, chira, khoi, lawa, muri, bread, bakery products, suji, maida, jowar products, besan, other gram products, cold and hot beverages, outside cooked meals including snacks, prepared sweets, namkeen, chips, pickles, sauce, jams, jelly, ice cream, biscuits, chocolates

^h^Spices include all spices like Ginger, garlic, jeera, dhania, turmeric, black pepper, dry chillies, tamarind, curry powder, oilseeds and other spices

Data not available in NSS on lard

- Below 1%

**Table 3:** Daily per capita caloric intake comparison by food groups – across regions

	EAT-Lancet			Regions			
		North	South	East	West	North East	Central
Whole grains^a^	811 (32%)	1218 (1211, 1226) (54%)	1115 (1106, 1124) (51%)	1346 (1335, 1358) (61%)	983 (972, 994) (46%)	1457 (1423, 1491) (68%)	1302 (1286, 1318) (59%)
Potato and Cassava	39 (2%)	76 (75, 77) (3%)	14 (14, 15) (1%)	106 (104, 107) (5%)	33 (32, 34) (2%)	53 (52, 55) (2%)	45 (44, 46) (2%)
Vegetables	78 (3%)	56 (55, 57) (2%)	55 (54, 56) (3%)	57 (56, 57) (3%)	59 (57, 61) (3%)	54 (53, 55) (3%)	51 (50, 52) (2%)
Fruits^b^	126 (5%)	30 (29, 31) (1%)	45 (44, 46) (2%)	24 (22, 25) (1%)	42 (40, 43) (2%)	31 (29, 34) (1%)	22 (21, 24) (1%)
Dairy Foods	153 (6%)	247 (242, 251) (11%)	131 (129, 133) (6%)	87 (84, 90) (4%)	159 (155, 163) (7%)	50 (47, 52) (2%)	115 (109, 120) (5%)
Protein sources	726 (29%)	99 (98, 100) (4%)	203 (201, 206) (9%)	107 (105, 109) (5%)	150 (146, 153) (7%)	123 (119, 126) (6%)	113 (110, 115) (5%)
All Animal source proteins	151 (6%)	11 (10, 11) -	51 (49, 52) (2%)	36 (35, 37) (2%)	21 (20, 22) (1%)	55 (54, 57) (3%)	11 (10, 12) (1%)
Beef and lamb	15 (1%)	3 (3, 3) -	5 (5, 6) -	3 (3, 4) -	4 (3, 4) -	5 (5, 6) -	1 (1, 1) -
Pork	15 (1%)	0 (0, 0) -	0 (0, 0) -	0 (0, 0) -	0 (0, 0) -	5 (5, 5) -	0 (0, 0) -
Poultry (Chicken & other)	62 (2%)	2 (2, 2) -	14 (14, 15) (1%)	6 (6, 7) -	6 (5, 7) -	8 (7, 9) -	4 (4, 4) -
Eggs	19 (1%)	3 (3, 4) -	13 (13, 14) (1%)	8 (8, 8) -	6 (6, 6) -	12 (12, 13) (1%)	3 (2, 3) -
Fish	40 (2%)	1 (1, 1) -	16 (15, 16) (1%)	16 (16, 17) (1%)	4 (3, 4) -	22 (22, 23) (1%)	2 (2, 2) -
Legumes3	426 (17%)	85 (84, 86) (4%)	101 (99, 102) (5%)	68 (66, 70) (3%)	106 (103, 108) (5%)	64 (62, 67) (3%)	91 (89, 93) (4%)
Tree nuts4	149 (6%)	2 (2, 3) -	51 (50, 52) (2%)	3 (3, 3) -	22 (21, 23) (1%)	2 (2, 3) -	10 (9, 11) -
Added fats	450 (18%)	225 (218, 231) (10%)	218 (215, 222) (10%)	183 (180, 186) (8%)	325 (316, 333) (15%)	156 (153, 160) (7%)	212 (206, 217) (10%)
Palm Oil^e^	60 (2%)	61 (56, 65) (3%)	161 (158, 164) (7%)	32 (30, 33) (1%)	257 (247, 266) (12%)	16 (15, 18) (1%)	162 (157, 168) (7%)
Unsaturated Fats^f^	354 (14%)	147 (145, 149) (7%)	54 (51, 58) (2%)	149 (147, 152) (7%)	56 (52, 61) (3%)	138 (135, 141) (6%)	41 (38, 44) (2%)
Dairy Fats	0 (0%)	16 (15, 18) (1%)	2 (2, 2) -	1 (1, 1) -	11 (10, 12) (1%)	1 (1, 1) -	8 (7, 8) -
Lard	36 (1%)	–	–		–	–	–
All sweeteners	120 (5%)	133 (130, 137) (6%)	92 (91, 93) (4%)	64 (63, 65) (3%)	137 (134, 140) (6%)	65 (64, 67) (3%)	112 (109, 115) (5%)
Processed Food^g^		172 (166, 177) (8%)	283 (272, 294) (13%)	228 (221, 235) (10%)	235 (225, 245) (11%)	176 (166, 186) (8%)	212 (193, 231) (10%)
Spices^h^		29 (29, 30) (1%)	55 (54, 56) (3%)	29 (28, 29) (1%)	39 (38, 40) (2%)	20 (20, 21) (1%)	33 (32, 33) (2%)
**Total**	2503	2259	2171	2211	2124	2158	2198

Mean values in kcal reported with 95% confidence intervals in parenthesis. Second row of each cell reports the mean value as a % of daily total calorie consumption. NSS-CES sampling weights were applied in calculations

Lighter font represents subgroups within major food groups

MPCE (Monthly Per Capita Expenditure) signifies classification of population by economic level. Highest and lowest MPCE refers to the richest and poorest 5% of the population respectively

^a^Whole grains consist of cereals like rice, wheat, wheat flour and other cereals like jowar, bajra, maize, barley, millets and ragi

^b^Fruits consists of all fruits and raisins, dates and other dried fruits

^c^Legumes consists of all pulses and groundnuts,

^d^Tree nuts consist of coconut, coconut green and coconut-*copra* (dried), cashew nuts, walnuts and other nuts

^e^Palm oil contains vanaspati, refined and edible oil

^f^Unsaturated oil contains mustard, groundnut and coconut oil

^g^Processed Food includes rice products, chira, khoi, lawa, muri, bread, bakery products, suji, maida, jowar products, besan, other gram products, cold and hot beverages, outside cooked meals including snacks, prepared sweets, namkeen, chips, pickles, sauce, jams, jelly, ice cream, biscuits, chocolates

^h^Spices include all spices like Ginger, garlic, jeera, dhania, turmeric, black pepper, dry chillies, tamarind, curry powder, oilseeds and other spices

Data not available in NSS on lard

- Below 1%

The average calorie intake from both plant and animal-based foods rich in protein is low in both rural and urban areas, across different income classes, and different parts of India. The same is true for the consumption of fruits and vegetables.

Interestingly, calorie shares of different food groups are similar between rural and urban households of similar per capita monthly expenditure levels. This is consistent with the results from a household survey in the state of Punjab that showed no significant rural-urban differences in diets [14].

The poorest 5% of the population in India consumes less than the suggested calories for all food groups except whole grains, tubers and starchy vegetables, and processed food. There is little rural-urban variation within this group.

### Cereals and starchy vegetables

EAT-Lancet Commission recommends that about one-third (811 kcals) of the total daily calorie intake should come from whole grains. The average Indian households get almost half (47%) of their total calories from whole grains and the calorie share of cereals is as high as 70% for the poorest rural households (Tables 2, 3 and 4).

**Table 4:** Difference in calories consumed in India and recommended by the EAT-Lancet reference diet for Whole grains and Tubers and starchy vegetables

Total caloric intake (kcal)	Whole grains	Tubers and starchy vegetables
Urban India	218 (212, 223)	12 (12, 13)
Rural India	464 (458, 471)	24 (23, 25)
Highest MPCE -Urban	53 (20, 86)	17 (13, 21)
Highest MPCE -Rural	536 (481, 592)	19 (14, 23)
Lowest MPCE- Urban	258 (236, 280)	17 (14, 20)
Lowest MPCE -Rural	348 (321, 374)	18 (15, 22)
North India	407 (400, 415)	37 (36, 38)
South India	304 (295, 313)	−25 (−25, −24)
East India	535 (524, 547)	67 (65, 68)
West India	172 (161, 183)	−6 (−7, −5)
North East India	646 (612, 680)	14 (13, 16)
Central India	491 (475, 507)	6 (5, 7)

Values in kcal. 95% confidence interval in parenthesis. NSS-CES sampling weights were applied in calculations

Whole grains consist of cereals like rice, wheat, wheat flour and other cereals like jowar, bajra, maize, barley, millets and ragi

### Protein sources

**Fig. 1:**
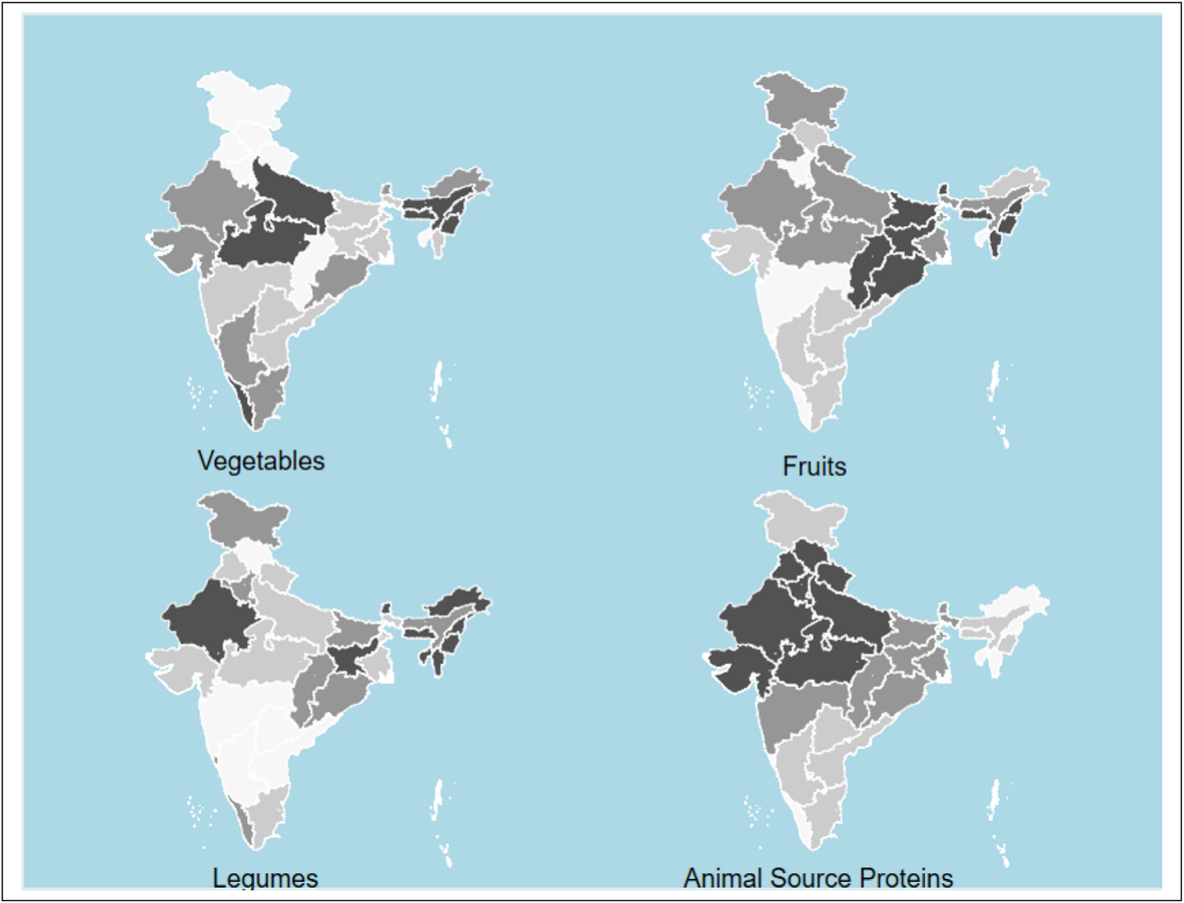
Caloric intake deficit* of vegetables, fruits, legumes and animal source proteins compared to reference diet. Animal source proteins include chicken, other poultry, eggs, lamb, beef, pork and fish. Darker colour depicts higher calorie difference between actual consumption and reference diet. Maps were generated using STATA statistical software version 15.0. *Difference between actual consumption and the daily per capita calorie intake suggested by the EAT-Lancet reference. State level caloric intake calculated using population weights for rural and urban populations. Values for Telangana same as that for Andhra Pradesh. NSS-CES sampling weights were applied in calculations

**Fig. 2:**
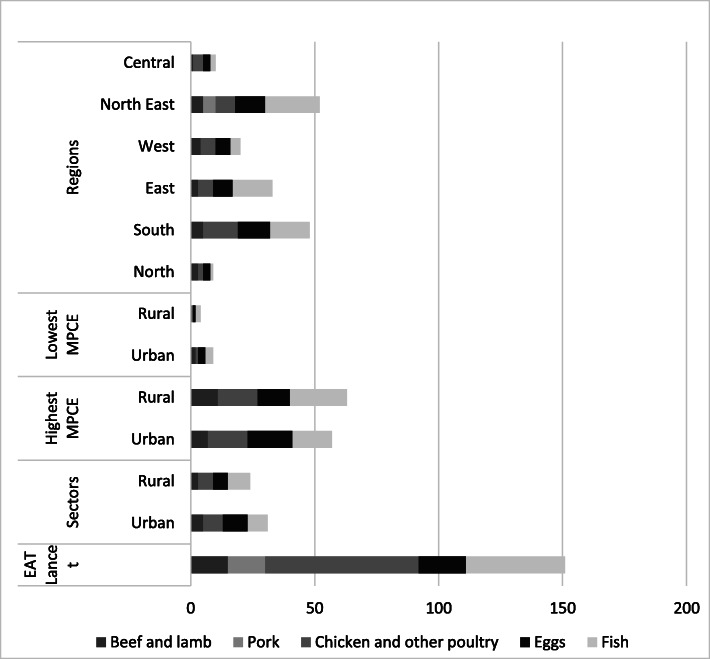
Caloric intake from various animal-based protein sources. Values in kcal. NSS-CES sampling weights were applied in calculations

When compared to the reference diet, low caloric intake from protein sources (both plant and animal-based) is common across all sectors, regions, and income groups of India (Tables 2, 3, and 4 and Figs. 1 and 2). The deficit is more in rural areas where only 6% of the total caloric intake comes from protein sources compared to 29% in the EAT-Lancet diet (Table 2).

Even for the richest 5% of India’s population, calories from protein sources is less than half of the 726 kcal in the reference diet. The poorest Indians get below 130 kcal per day (less than 20% of the recommendation) from protein sources. Among different regions of India, people in the North-east consume the lowest quantities of legumes (Fig. 1 and Table 5).

**Table 5:** Difference in calories consumed in India and proposed by EAT-Lancet reference diet for Legumes and Tree Nuts

Total caloric intake (kcal)	Legumes	Tree nuts
Urban India	− 331 (−331, − 330)	−128 (−128, −127)
Rural India	− 342 (− 343, − 341)	−135 (−135, −134)
Highest MPCE -Urban	− 304 (− 309, −299)	−102 (−106, −98)
Highest MPCE -Rural	− 283 (−294, − 273)	−97 (− 101, −93)
Lowest MPCE- Urban	− 365 (− 367, − 363)	− 145 (− 146, − 145)
Lowest MPCE -Rural	− 371 (− 373, − 368)	−148 (−148, −147)
North India	−341 (− 342, − 340)	−147 (− 147, −146)
South India	− 325 (− 327, − 324)	− 98 (−99, − 97)
East India	−358 (− 360, −356)	− 146 (− 146, − 146)
West India	− 320 (−323, −318)	− 127 (− 128, − 126)
North East India	− 362 (− 364, − 359)	− 147 (− 147, − 146)
Central India	−335 (− 337, − 333)	− 139 (− 140, − 138)

Values in kcal. 95% confidence interval in parenthesis. NSS-CES sampling weights were applied in calculations

Legumes consists of all pulses and groundnuts,

Tree nuts consists of coconut, coconut-green, coconut-dried, cashew nuts, walnuts and other nuts

The rich in India get fewer calories from cereals and more calories from fruits, vegetables, animal source proteins, and fats compared to the poor. (Tables 2 and 3).

The EAT-Lancet reference diet advocates consumption of only “low to moderate” quantities of seafood and poultry and “no to low” amounts of red meat or processed meat. Animal-source proteins constitute 6% of the total caloric intake in the reference diet. Indian diets, across different types of households, have a lower share of calories from animal-based foods. Consumption of animal-based foods is relatively higher in South India and the North-Eastern region. The richest households in all parts of India consume more than average quantities (or calories) of meat. (Figs. 1 and 2). Consumption of red meat (beef, lamb and pork) is low in India (Table 2, 3 and Fig. 2).

### Fruits and vegetables

**Fig. 3:**
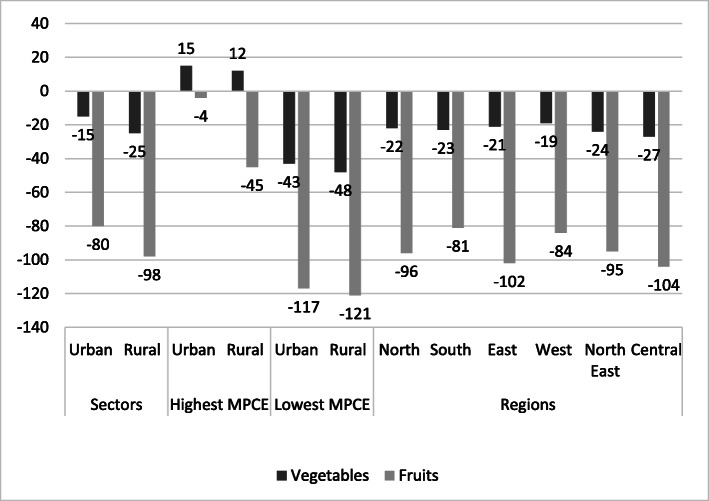
Difference in caloric intake from Vegetables and Fruits between EAT-Lancet reference diet and Indian diets. Caloric intake calculated as total kcal/person/day. NSS-CES sampling weights were applied in calculations

A diverse diet is a healthy diet [15, 16]. The reference diet is largely plant-based with fruits and vegetables accounting for 204 kcal (8%) of the daily calorie intake. Most Indians, except the richest ones in urban areas, do not consume enough fruits. The average consumption of calories from fruits is less than 40% of what EAT-Lancet recommends (Table 2, 3 and Fig. 3). The difference in caloric intake from vegetables is smaller than what it is for fruits, but under-consumption of vegetables is also common across all but the richest households in rural and urban areas (Fig. 3).

### Oils and fats

**Fig. 4:**
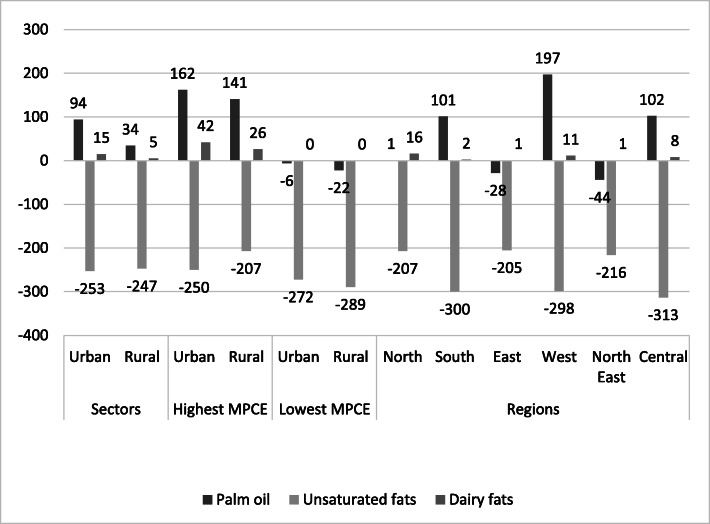
Caloric intake difference for added fats between Indian diets and proposed EAT-Lancet reference diet. Indian diets across sector, MPCE fractile classes and regions. Values in kcal. NSS-CES sampling weights were applied in calculations

Indians get fewer calories from added fats than what is recommended in the reference diet (Tables 2 and 3). This is despite an increase of 3.5% in the consumption of oils and fats between 1993 and 94 and 2011–12 in both rural and urban areas as per NSS reports. Figure 4 shows that Indians, in general, consume less of the healthier oils & fats and more of the unhealthier saturated fats like palm oil.

Palm oil is high in unhealthy saturated fat and is the chief ingredient of the widely consumed *vanaspati* (partially hydrogenated vegetable oil) in India.

The consumption of *vanaspati* is known to have risen by 51% between 1993 and 94 and 2011–12 and it is widely used for cooking at home, in restaurants, by street vendors, and in the preparation of processed foods [17]. Figure 4 also shows that the highest caloric consumption of palm oil is among the highest income groups. Among different parts of India, consumption is highest in western India.

It should be noted that the consumption of fat in India might be higher than reflected in this data as the fat content of packaged food and meals consumed outside of the home is not accounted for.

### Processed food

EAT-Lancet commission recommends the consumption of only small amounts of processed food. Processed food is not even a separate food group in the reference diet. We report the consumption of processed food as a separate group in this paper because of its high and rising consumption in India. In the 68th round of NSS-CES, bread, bakery products, *suji* (semolina), *maida* (refined wheat flour), cold and hot beverages, outside cooked meals including snacks, prepared sweets, savouries (*namkeens*), chips, pickles, sauce, jams, jelly, ice cream, biscuits, chocolates have been included in the category of processed food along with meals consumed outside of the home. These types of foods, normally high in sugar, salt, saturated fats, and processed flour, are considered unhealthy and often linked to obesity/overweight and NCDs [2, 18–23].

**Fig. 5:**
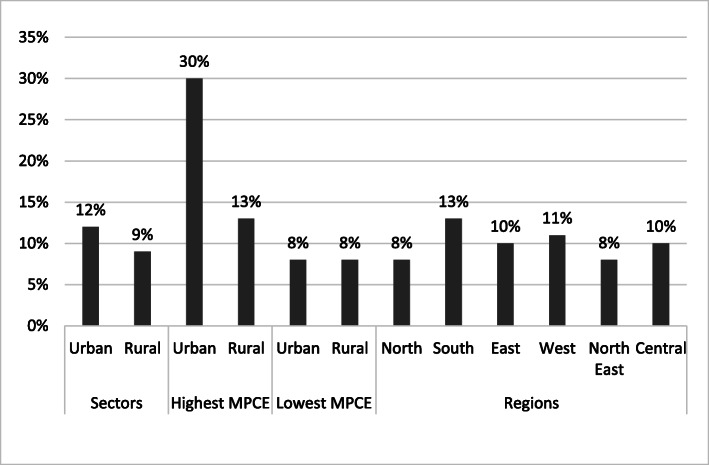
Proportion of daily per capita caloric intake from processed food in India. NSS-CES sampling weights were applied in calculations

Processed food accounts for nearly 10% of the average total caloric intake in both rural and urban India (Table 2, 3 and Fig. 5). Urban households in the highest income group consume almost 30% of their total daily calories from processed food (Fig. 5). Among different regions of India, the calorie share of processed food is highest in Southern Indian and the lowest in North-Eastern and Northern India (Tables 2 and 3).

Spices like ginger, garlic, coriander powder (*dhania)* and turmeric constitute 2% of total caloric consumption in rural and urban India.

## Discussion

Diets in India are unhealthy and very different in their composition from the EAT-Lancet reference diet or even diets recommended by the ICMR. In a recent paper, Hirvonen et al. argue that the EAT-Lancet reference diets are not affordable for much of the world’s (and South Asia’s) low-income population. In South Asia, the reference diet will cost more than 60% of the mean daily per capita household income and cost more than 1.5 times the least-cost nutritionally adequate diet [24]. Hirvonen et al. also show that fruits, vegetables, and animal products are the most expensive among the major food groups across the world. Low affordability of healthy foods may be one of the reasons for their low consumption in India.

High subsidies on rice and wheat through the public distribution system and active management of the markets to keep market prices of cereals at low levels incentivize people to eat more rice and wheat.

Our analysis, however, shows that low affordability is not the only reason why Indian diets are so unhealthy. Even the richest 5% of households consume too little protein-rich food and too much processed foods. In rural areas, even the richest families eat more than recommended quantities of cereals and not enough fruits and vegetables. This points towards a lack of availability, accessibility, awareness, and acceptability as other major causes for the poor quality of diets.

Legumes are the main source of non-cereal plant protein in Indian diets. However, their consumption is low. The production of pulses has grown slower than the population, resulting in a steady decline in their per capita availability and consumption over the last five decades. Milk and other dairy products are the most common sources of animal protein in India. The majority of Indians now identify themselves as non-vegetarian [25]. Data from the National Family Health Survey conducted in 2015–16 in India supports this claim and indicates that only 20–30% population is vegetarian, having never had fish, chicken, meat, and eggs [25]. Yet, majority of the non-vegetarians report that they consume meat only occasionally. Meat production is projected to continue its fast growth at 3.1% p.a. up to 2023, with poultry dominating meat production. Per capita fish consumption is also expected to grow at 0.9% p.a. to reach 6.8 kg in 2023 [26]. This indicates that while the consumption of animal products is rising in India with rising incomes and urbanization, it is still significantly below the world average.

Rising imports of cheap palm oil have led to an increase in their consumption over the years—both in-home cooking and in the form of processed foods in India. Before 1992, edible oil was on the negative list and imports were disallowed. With liberalization, Palm oil is the largest food import in India [17, 27] and more than half of domestic consumption of oil comprises imports.

Indian diets are unhealthy also because healthier calories are more expensive and their inflation is rising faster than cereals and edible oils [27, 28].

Overall, dietary risks were responsible for 22% of all deaths and of all DALYs amongst adults [29]. Diets low in fruits, vegetables, and whole grains but high in salt, sugar, and fat (which constitute dietary risk) are also responsible for India’s increasing disease burden [23].

Our comparison of Indian diets with EAT-Lancet recommendations has several limitations. First, NSS-CES (and other consumption expenditure surveys) tend to underestimate total calorie consumption, especially calories consumed from meals taken outside home and from processed foods [30]. These differences are bigger for richer households. Second, NSS-CES uses 30-days and 7-days recall data when 24-h recall data is considered more accurate. There may be systematic errors due to a longer recall period in this data set. Third, there are significant gender and age-related differences in the diets within Indian households. Women eat a poorer diet than men in their families [31, 32]. However, the NSS-CES data does not capture these intra-household differences as it only collects aggregate household consumption information. Finally, the latest available NSS-CES is already 8 years old and pre-dates the implementation of the National Food Security Act (NFSA)-2013 that led to a significant increase in the public distribution of highly subsidized rice and wheat, potentially affecting dietary patterns of the poorest two-thirds of Indian households. Therefore, recent changes in the dietary pattern and behaviour cannot be analysed.

Another limitation of this study is due to the nature of the EAT-Lancet reference diet itself. It pertains to a typical adult person, considers only a limited set of nutrients, and ignores differences in bioavailability across different food groups [24]. These limitations suggest the need for better, more disaggregated dietary data with a shorter recall period and more research to develop a better understanding of different nutritional requirements of different groups of people.

Two factors help with the reliability of estimates of dietary intake in this paper. First, NSS-CES visits each district 4 times in a year. The repeat visits increase the chance of capturing the usual consumption pattern of households and minimize any seasonal variations. Second, NSS-CES has a large sample size. Any underlying uncertainty is thus likely to be a function of measurement error rather than sample size. Our comparison of Indian diets with the EAT Lancet reference diets still provides important insights.

Promoting healthy diets requires a major policy reset in India. India’s food policies and budget allocations are focused almost entirely on ensuring the affordability of rice and wheat. Almost all food subsidy—both for farmers and consumers—is spent on promoting rice and wheat production and consumption. Trade policies work to ensure low prices for sugar and palm oil. Since the policy incentivizes farmers to grow more rice, wheat, and sugarcane, the production of healthier foods, like pulses, fruits, and vegetables is lower than what it would be without these policy distortions. Food policies need readjustments to ensure the availability of healthier foods at affordable prices.

Moreover, raising consumer awareness about the need for dietary diversification can encourage families to switch to healthier foods. The consumer subsidy on food should shift away from rice and wheat to healthier options. Cash transfers in combination with an intensive communication campaign can accelerate the shift to healthier diets.

## Conclusion

The EAT-Lancet reference diet is described as a healthy and sustainable diet. Based on the analysis of consumption data, it is evident that Indian diets across urban-rural divide, regions, and income levels, deviate significantly from this reference diet and are far from being healthy for humans or the environment. Unhealthy diets are major contributors to persistently high levels of undernutrition (including micronutrient deficiencies) and rising levels of overweight and obesity in India.

As discussed in the paper, a shift to healthier diets will also require a change in production patterns. Currently, India produces too much rice and sugarcane and too little coarse cereals, pulses, fruits, and vegetables. Rice and sugarcane have big environmental footprints. Both are highly water intensive. Wet rice fields also emit methane, a powerful greenhouse gas. Moreover, rice farmers in many states of India burn rice residues emitting carbon dioxide and particulate matter creating severe air pollution. The shift in cropping patterns towards coarse cereals and pulses will make India’s food systems not only healthier but also environmentally more sustainable. Making food systems healthier and environmentally more sustainable requires public health and nutrition policies addressing malnutrition. It also requires agriculture, trade, and consumer awareness policies that can address the accessibility, acceptability, and affordability of healthier dietary options. A transformation of the Indian food system is much needed for both human health and environmental sustainability.

## Data Availability

The data used in this article is from the Household Consumer Expenditure, National Sample Survey, 68th Round, which contains anonymised data in the public domain. The survey is undertaken by the National Sample Survey Office (NSSO) of the Ministry of Statistics and Programme Implementation of the Government of India and is available from the Indian Council of Social Science Research (ICSSR): http://www.icssrdataservice.in/datarepository/index.php/catalog/135. Unit-level data is also available at a nominal price from the Ministry of Statistics and Programme Implementation of India. As the analysis is based on publicly available secondary data, ethics approval or consent to participate are not applicable.

## Abbreviations

NSS-CES: National Sample Survey-Consumer Expenditure Survey
NFHS: National Family Health Survey
DALY: Disability-adjusted life year
NCDs: Non-communicable diseases
WHO: World Health Organization
MPCE: Monthly per capita expenditure
RDA: Recommended Dietary Allowances RDAs
ICMR: Indian Council of Medical Research

## Footnotes

1 Details about sampling, data collection, questionnaires, as well as reports of the NSS can be found on the website of the Indian Council of Social Science Research (ICSSR): http://www.icssrdataservice.in/datarepository/index.php/catalog/91.

2 The Indian Council of Medical Research (ICMR) recommends per-person per-day calorie norms of 2400 kcal for rural areas and 2100 kcal for urban areas.
